# Calculation of Integral Indicators of the Metabolic Status and Filtration Function of Kidneys in Patients with a Combat Mine-Explosive Injury who Lost Significant Body Weight due to Limb Amputation

**DOI:** 10.15388/Amed.2025.32.1.2

**Published:** 2025-02-18

**Authors:** Nataliia Sydorova, Kateryna Kazmirchuk, Oleh Kolisnyk, Vira Tsaralunha, Yuliia Kobirnichenko, Liudmyla Sydorova

**Affiliations:** 1 Department of Military General Practice and Family Medicine, Ukrainian Military Medical Academy, Kyiv, Ukraine; 2 Nephrology Clinic with Wards for Endocrinological Patients, National Military Medical Clinical Center “Main Military Clinical Hospital”, Kyiv, Ukraine; 3 Medical and Diagnostic Department, National Military Medical Clinical Center “Main Military Clinical Hospital”, Kyiv, Ukraine; 4 Clinic of Laboratory Diagnostics, National Military Medical Clinical Center “Main Military Clinical Hospital”, Kyiv, Ukraine

**Keywords:** limb amputation, body mass index, correction, glomerular filtration rate, hyperfiltration, traumatic amputation, combat trauma, galūnės amputacija, kūno masės indeksas, korekcija, glomerulų filtracijos greitis, hiperfiltracija, trauminė amputacija, kovinė trauma

## Abstract

**Background:**

The aim of this retrospective cohort study was to investigate the possibility of optimizing the calculation of integral indicators of the metabolic status and filtration function of the kidneys in patients with combat mine-explosive injuries who have lost significant body weight due to limb amputation.

**Methods:**

Data from 81 servicemen (all males) with combat mine-explosive limb injuries, including those with isolated severe limb injuries (Group 1, n=34) or traumatic amputations (Group 2, n=47), were analyzed. We assessed demographic indicators and the glomerular filtration rate (GFR), calculated according to the generally accepted formulas with correction for lost body parts in the amputees.

**Results:**

The proportion of amputees with obesity as measured by the body mass index in Group 2 without correction for the lost body parts was significantly lower than that in Group 1 and Group 2 adjusted for the lost body parts (2.12% versus 17.65% and 21.28%, respectively, p<0.05 and p<0.01), but it was similar in Group 1 and Group 2 after body weight adjustment for the lost body parts of the amputees (p>0.05). The mean level of serum creatinine in the patients in Group 2 was significantly lower than that in Group 1 (75.19±11.62 vs. 90.93±37.12 μmol/L, respectively, p=0.0206). The estimated GFR according to the *Cockcroft and Gault formula* (CGF) with adjusted for the lost body part body weight was significantly greater in Group 2 than that in Group 1 (143.63±42.58 and 123.92±26.34 mL/min/1.73m^2^, respectively, p=0.0379) as well as GRF according to CGF corrected for the *body surface area* (BSA) (131.59±39.94 and 106.17±21.88 mL/min/1.73m^2^, respectively, p=0.0040). Only a few individuals had a moderate decrease in GFR according to CGF or CGF adjusted for BSA, but glomerular hyperfiltration was suspected in a significant number of patients, specifically, 23.53% and 17.65% of the patients in Group 1, respectively, and in 29.79% and 36.17% of the patients in Group 2, respectively (even 51.6% according to CGF corrected for BSA in Group 2 adjusted for the lost body parts).

**Conclusions:**

In amputees, it is necessary to calculate their body weight considering the lost body part for an adequate assessment of their metabolic status. To calculate GFR, it is advisable to use CGF considering the lost body parts with or without adjustment for BSA to avoid the possibility of underestimating GFR calculated by other formulas. Special control is necessary for patients with hyperfiltration suspected by CGF, since this sign can be a predictor of future metabolic disorders.

## Abstract (it)

**Įvadas:**

Šio retrospektyvaus kohortinio tyrimo tikslas – ištirti galimybę optimizuoti pacientų, patyrusių kovinį minosvaidžio sužeidimą ir dėl galūnių amputacijos netekusių daug kūno svorio, inkstų metabolinės būklės ir filtravimo funkcijos integralių rodiklių skaičiavimą.

**Metodai:**

Buvo analizuojami 81 kario (visi vyrai), patyrusio kovinius minosvaidžio sprogstamuosius galūnių sužalojimus, įskaitant tuos, kuriems buvo nustatyta pavienių sunkių galūnių sužalojimų (1 G grupė, n = 34) arba trauminės galūnių amputacijos (2 G grupė, n = 47), duomenys. Vertinti demografiniai rodikliai ir glomerulų filtracijos greitis (GFR), apskaičiuotas pagal visuotinai pripažintas formules ir atliekant korekciją dėl amputuotų kūno dalių praradimo.

**Rezultatai:**

Amputuotų asmenų, kuriems nustatytas kūno masės indeksas, dalis 2 grupėje be korekcijos dėl prarastų kūno dalių buvo reikšmingai mažesnė nei 1 ir 2 grupėse, pakoreguotose dėl prarastų kūno dalių (2,12 %, palyginti su 17,65 % ir 21,28 %, atitinkamai p < 0,05 ir p < 0,01), tačiau ji buvo panaši 1 ir 2 grupėse, pakoregavus amputuotų asmenų kūno masę dėl prarastų kūno dalių (p > 0,05). Vidutinis kreatinino kiekis serume 2 Gg grupės pacientų buvo reikšmingai mažesnis nei 1 Gg grupės pacientų (atitinkamai 75,19±11,62 vs. 90,93±37,12 μmol/l, p = 0,0206). Įvertinta GFR pagal Kakrofto ir Golio formulę (CGF), pakoreguota atsižvelgiant į prarastą kūno dalies kūno svorį, 2 grupės pacientų buvo reikšmingai didesnė nei 1 grupės pacientų (atitinkamai 143,63±42,58 ir 123,92±26,34 ml/min/1,73m^2^, p = 0,0379), taip pat GRF pagal CGF, pakoreguota atsižvelgiant į kūno paviršiaus plotą (BSA) (atitinkamai 131,59±39,94 ir 106,17±21,88 ml/min/1,73m^2^, p = 0,0040). Tik keliems asmenims GFR pagal CGF arba CGF, pakoreguotą pagal BSA, sumažėjo vidutiniškai, tačiau glomerulų hiperfiltracija buvo įtariama nemažai daliai pacientų, konkrečiai – 23,53 % ir 17,65 % pacientų atitinkamai 1 G grupės ir 29,79 % ir 36,17 % pacientų 2 G grupės (net 51,6 % pagal CGF, pakoreguotą pagal BSA, 2 G grupėje, pakoreguotą pagal prarastas kūno dalis).

**Išvados:**

Amputuotų pacientų metabolinei būklei tinkamai įvertinti būtina apskaičiuoti kūno svorį atsižvelgiant į prarastą kūno dalį. Skaičiuojant GFR, patartina naudoti CGF, atsižvelgiant į prarastas kūno dalis, koreguojant pagal BSA arba nekoreguojant, kad būtų išvengta galimybės nepakankamai įvertinti pagal kitas formules apskaičiuotą GFR. Speciali kontrolė būtina pacientų, kuriems pagal CGF įtariama hiperfiltracija, nes ji gali būti medžiagų apykaitos sutrikimų prognostinis požymis.

## Introduction

The management of patients with combat limb injuries who have undergone amputation is a relatively new and generally insufficiently studied issue for Ukraine [1]. Approaches to the therapeutic support of this contingent after a surgical intervention constitute a special group of questions considering this problem. Is it necessary to adjust the dose of drugs in cases where it depends on the body weight in patients who have lost a significant part of it due to traumatic amputation? Do amputees have peculiarities in the pharmacokinetics of drugs, especially those with a narrow therapeutic index or an unfavorable toxicity profile? How should the *body mass index* (BMI) be calculated in this contingent to assess the metabolic status and the *glomerular filtration rate* (GFR) as a measure of the renal function? What should we consider if a dose correction of some medicine is necessary for a patient with a traumatic amputation?

In the clinical practice, GFR is usually estimated by using endogenous (serum) markers with a sufficient accuracy (creatinine, urea, cystatin C). The most accurate ‘gold standard’ method for GFR determination is the use of exogenous markers (inulin, sodium iothalamate, iohexol); however, these methods are expensive and are not deemed standard in the clinical practice [2].

Several formulas have been proposed for calculating GFR based on the creatinine clearance, which is the most widely available indicator in the real clinical practice: the *Cockcroft and Gault formula* (CGF, which considers the age, sex, and body weight data [3]), the *Modification of Diet in Kidney Diseases formula* (MDRD, which considers the age, sex, and race [4]), and the *Chronic Kidney Disease Epidemiologists Collaboration formula* (CKD-EPI), which also considers the age, sex, and race [5,6].

Nevertheless, there is a lack of information about the need to adjust the dose of drugs administered to military personnel after traumatic limb amputation. Several methods for calculating GFR in amputees have been discussed [7–12]. Himes [7] developed an equation to estimate the body mass index in amputees, whereas other authors, e.g., Spain et al. [12], debated the idea to control the metabolic state of the amputee by assessing the fat distribution. The corrected ‘ideal’ body weight assessment in amputees, meanwhile, is the most popular because of its simplicity and availability [8–11,13,14]. Therapeutic monitoring of drugs is recommended when prescribing drugs whose dose depends on the renal function (aminoglycosides, vancomycin, etc.) or BSA (chemotherapeutic drugs, carboplatin, 6-mercaptopurine, etc.) [13]. The same authors also recommend calculating the ‘ideal’ body weight for amputees, considering the percentage of the lost body weight by using the proportions of body parts described by Osterkamp [13,14].

**The aim** of this research was to study the possibility of optimizing the calculation of integral indicators of the metabolic status and filtration function of the kidneys in patients with combat mine-explosive limb injuries who have lost significant body weight due to limb amputation.

## Methods

This retrospective cohort study is a fragment of the study ***P****redictors of de novo p****A****thology of in****T****ernal o****R****gans* ***I****n c****O****mba****T****ants with severe combat trauma and limb amputations* (PATRIOT), which is ongoing at the health care institutions of the Ministry of Defense of Ukraine since 2024 [1]. The overall objective of the PATRIOT study is to determine the therapeutic aspects of the management of military personnel who have undergone limb amputation due to combat trauma. In turn, the PATRIOT study is a part of the research of the Ukrainian Military Medical Academy on the topic “Scientific Substantiation of the Standardization of the System of Medical Support of the Armed Forces of Ukraine in Different Operating Conditions”, code *Standard*, State Registration No. 0116U002816.

To achieve the goal of the study, eighty-one servicemen (all men) who received an isolated mine-explosive limb injury were selected from hospital registries.

The criteria for inclusion in the study were the presence of a mine-explosive limb injury up to one-week prior, the male sex, age under 50 years, and the absence of a pathology of internal organs, particularly hypertension, obesity, diabetes, and kidney diseases.

The patients were allocated to two groups depending on the presence of limb amputation: Group 1 (n=34), which included servicemen who received only an isolated mine-explosive limb injury (without amputation); and Group 2 (n=47), which included combatants with limb amputations because of an isolated mine-explosive limb injury.

We assessed the demographic and clinical parameters, including the age, height, body weight, BMI, blood pressure, and serum creatinine levels. For Group 2, the parameters whose calculation required a correction for the body weight or BSA were presented by us as ‘Group 2 adjusted’. In this adjusted Group 2, we calculated the body weight and BMI with a correction for the body part loss due to amputation.

The GFR value was calculated according to the generally accepted formulas which do not include the body weight (CKD-EPI [5], MDRD [4]) and according to CGF [3,16], which is based on the body weight (CGF1) or normalized to the 1.73 m^2^ value of BSA (CGF2) [16–18] in Groups 1 and 2. In Group 2 adjusted, CGF1 and CGF2 were calculated with a correction for the body part loss due to amputation.

We used the adjusted body weight (BW_a_) for Group 2 adjusted CGF1. To calculate the BW_a_ value, we used the method described by Osterkamp [14] and currently used by different researchers [7–12] with the formula: BW_a_ = BW_m_ / (1 – *p*), where W_m_ is the measured body weight, and *p* is the proportion of the body weight that is missing due to amputation [19].

For BSA-based CGF2 calculation, we used online calculators with the automatic calculation of BSA, based on the body weight and height with the multiplication of the CGF parameters by 1.73/BSA [16].

We suspected GFR as renal hyperfiltration in the case of obtaining a GFR value >130 mL/min/1.73 m^2^ [20,21], and as decreased in the case of a GRF value of <90 mL/min/1.73 m^2^ [22].

The methods used in the study met the currently applicable ethical norms and standards. The research was carried out in accordance with the requirements of the Declaration of Helsinki of the World Medical Association “Ethical Principles of Medical Research with the Participation of a Person as a Research Object”, the *International Code of Medical Ethics* and the *Geneva Declaration*, as well as internal regulatory documents of the National Military Medical Clinical Hospital “Main Military Clinical Hospital” (Kyiv, Ukraine). Given the retrospective and non-interventional nature of the study, there was no need for the patients to provide informed consent. The research design was approved by the institution’s Ethics Committee and the Scientific and Methodical Bureau (Protocol No. 29, 26 February 2024). Although the calculation of GFR by several methods simultaneously was not a routine practice, and retrospective data from medical records contained only GFR values calculated by using the CKD-EPI equation, when approving the study protocol, a consensus was reached that any additional calculation of GFR by using several methods based on the already existing values does not violate the ethical principles of the retrospective non-interventional study design, since it does not have any harmful effect on the patient, but it is extremely necessary for the subsequent optimization of medical care for patients with traumatic amputation.

All the personal data of the patients were securely protected. Statistical analysis of the data was performed by the standard statistical package *STATISTICA 6.0* and *Microsoft Excel 2010* software by using parametric statistical methods. Indicators are presented as *percentages* (%) or *mean values* (M) ± *standard deviation* (SD). We used the method of alternative variation if the indicators were presented in % or a *two-sample t test* with different variances for the mean values. The value of p<0.05 was considered to indicate statistical significance.

## Results

**Figure 1:**
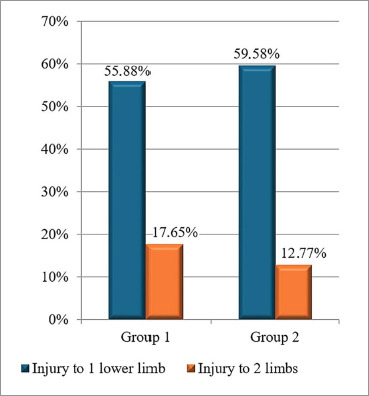
Proportion of patients with injury to one lower limb and injury to two limbs

All patients from both groups had experienced a mine-explosive combat injury with localization in most cases in the lower extremities: 55.88% of the patients in Group 1 and 59.58% of the patients in Group 2 (Figure 1). At the same time, 17.65% of the injured individuals in Group 1 and 12.77% of the combatants in Group 2 had lesions in two limbs. There was no significant difference between the groups in terms of the lesion location (p>0.05 for all patients).

**Figure 2:**
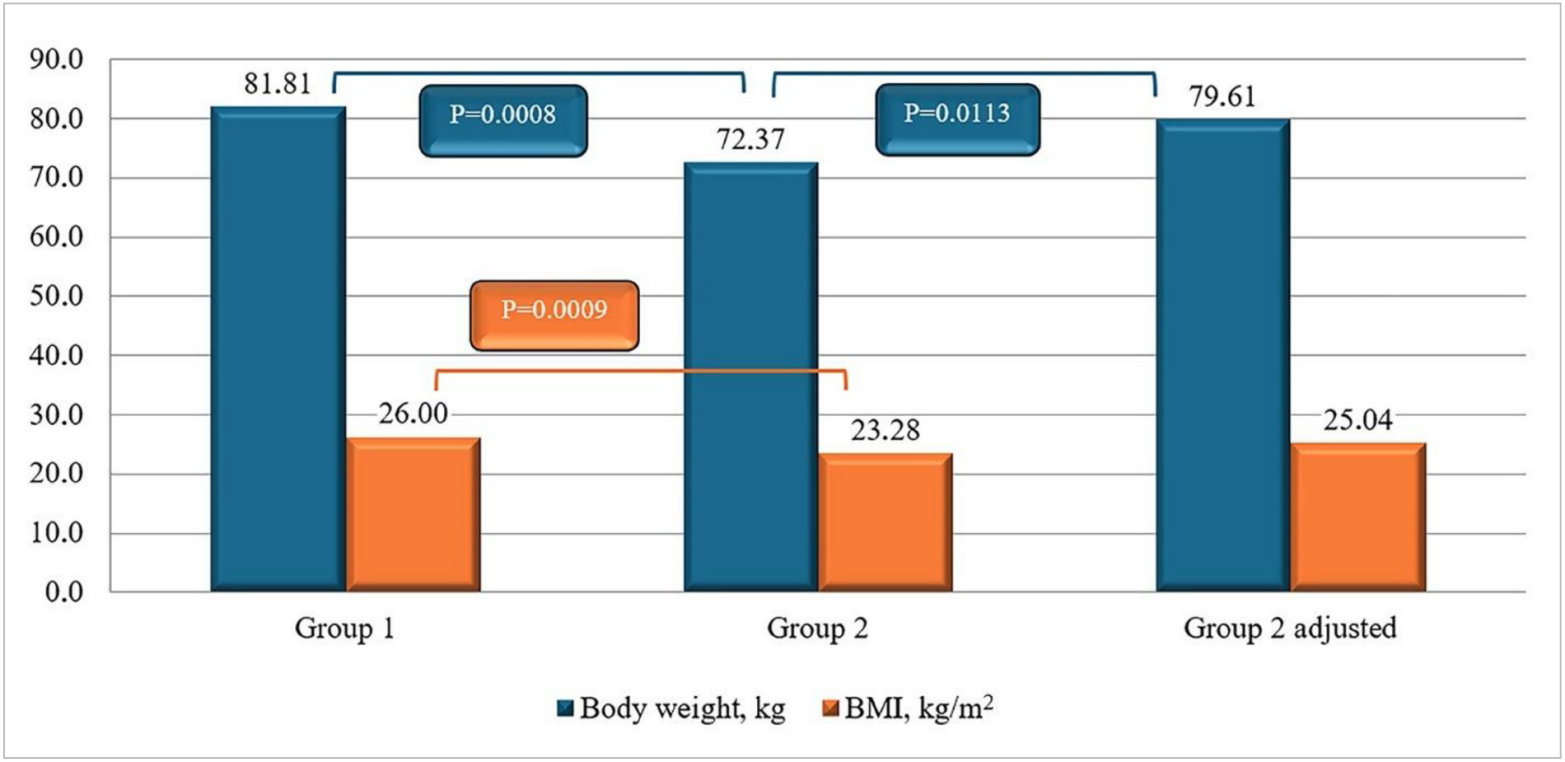
Body weight and BMI of military personnel who experienced a combat limb injury

The examined servicemen of both groups were of a similar age, and the average height values did not differ (Table 1). The mean body weight of the patients in Group 2 was expectedly lower than that of the patients in Group 1 (72.37±11.99 and 81.81±11.34 kg, respectively, p=0.0008); the mean uncorrected BMI was also significantly lower (23.28±3.49 and 26.00±3.25 kg/m^2^, respectively, p=0.0009). However, a correction for the lost body weight in the patients in Group 2 adjusted showed that, without amputation, the body weight and BMI in both groups would have been similar (p>0.05 for the differences between these indicators in Group 1 and Group 2 adjusted for both indicators; see Table 1 and Figure 2).

**Table 1:** Basic demographic data and main relevant clinical indicators of servicemen with combat limb injuries from their medical records

Indicator	Group 1 (n=34)	Group 2 (n=47)	P value
Age, years	39.93±9.93	35.35±9.63	>0.05
Height, m	1.78±0.08	1.77±0.06	>0.05
Body weight, kg	81.81±11.34	72.37±11.99	0.0008
BMI, kg/m^2^	26.00±3.25	23.28±3.49	0.0009
SBP, mm Hg	120.11±7.45	117.37±16.26	>0.05
DBP, mm Hg	77.50±6.72	75.65±7.44	>0.05
Creatinine, μmol/L	90.93±37.12	75.19±11.62	0.0206
CKD-EPI, mL/min/1.73 m^2^	97.92±18.73	100.10±26.82	>0.05

SBP – systolic blood pressure; DBP – diastolic blood pressure

**Figure 3:**
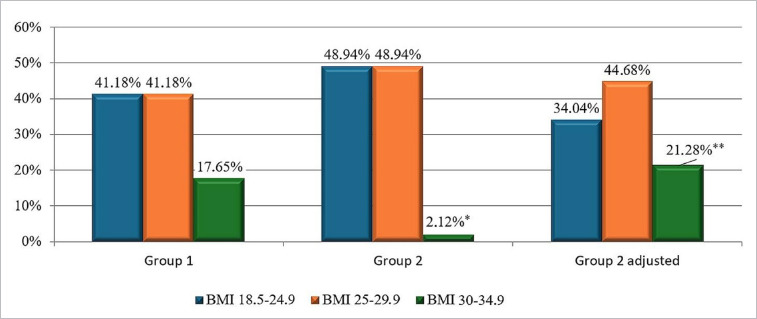
The structure of BMI categories of military personnel who experienced a combat limb injury. * – significant difference for indicators between Groups 1 and 2 (p<0.05**)**; **– significant difference for indicators between Group 2 and Group 2 adjusted (p<0.01**)**

The analysis of individual BMI indicators revealed that the proportion of patients with obesity in Group 2 without a correction for the lost body weight was significantly lower than that in Group 1 and Group 2 adjusted (2.12% versus 17.65% and 21.28%, respectively, p<0.05 and p<0.01) (Figure 3). At the same time, the proportion of patients in this weight category in Group 2 adjusted did not differ from that in Group 1. These data indicate the need to calculate the corrected (according to Elbarbry et al. [3]) body weight in amputees for an adequate assessment of their metabolic status.

The mean level of serum creatinine in the patients of Group 2 was lower than that in the patients of Group 1 (75.19±11.62 vs. 90.93±37.12 μmol/L, p=0.0206). The mean values of GFR calculated according to CKD-EPI (see Table 1) and MDRD did not differ significantly, but a higher GFR in amputees (as described in the literature) was not observed when calculated according to these formulas (see Table 2). There was no significant difference in the mean values of the creatinine clearance calculated in Groups 1 and 2 according to CGF1 and CGF2 (123.92±26.34 vs. 115.90±14.1 mL/min and 106.17±21.88 vs. 106.31±38.22 mL/min/1.73 m^2^, respectively, p for both indicators >0.05). When calculating the adjusted creatinine clearance in Group 2 according to CGF1 and CGF2, the average values significantly exceeded those in the patients in Group 1 and Group 2 (unadjusted) (see Table 2). These data confirm the information retrieved from scientific sources about the possibility of a decrease in the serum creatinine level and an increase in GFR in amputees, which can be ‘masked’ when calculating it according to the CKD-EPI and MDRD formulas [2,15,23,24].

**Table 2:** Results of GFR/creatinine clearance calculation in servicemen with a combat limb injury

Indicator	Group 1 (n=34)	Group 2 (n=47)	Group 2 adjusted (n=47)	P1 value	P2 value	P3 value
MDRD, mL/min/1.73m^2^	91.88±21.70	92.64±29.80		>0.05		
CGF1, mL/min	123.92±26.34	115.90±14.10	143.63±42.58	>0.05	0.0379	0.0077
CGF2, mL/min/1.73 m^2^	106.17±21.88	106.31±38.22	131.59±39.94	>0.05	0.0040	0.0078

P1 – difference in indicators between Groups 1 and 2; P2 – difference in indicators between Group 1 and Group 2 adjusted; P3 – difference in indicators between Group 2 and Group 2 adjusted

The analysis of individual GFR values revealed a moderate decrease when calculated according to the CRD-EPI and MDRD formulas (in 23.53–38.30% of the examined patients) without a significant difference between the indicators of Group 1 and Group 2 adjusted, while only single individuals had a moderate decrease in the creatinine clearance according to CGF1 and CGF2 (see Table 3). Such data once again draw attention to the possibility of underestimating the GFR value when calculated according to the CRD-EPI and MDRD formulas in the young population.

Interestingly, hyperfiltration was suspected in a significant number of patients when GFR was calculated according to CGF1 and CGF2: 23.53% and 17.65% of the patients in Group 1, 29.79 and 36.17% of the patients in Group 2 adjusted, respectively. When corrected for the lost body part, the proportion of amputees with renal hyperfiltration based on the creatinine clearance calculated by the CGF with a correction for BSA even exceeded 50% (p value for comparison with Group 1 <0.001, with Group 2 unadjusted <0.05). At the same time, cases of hyperfiltration were isolated when we calculated GFR according to CRD-EPI and MDRD (Table 3).

**Table 3:** Analysis of individual indicators of GFR/creatinine clearance in servicemen who experienced a combat limb injury

Technique	GRF value	Group 1 (n=34)		Group 2 (n=47)		Group 2 adjusted (n=47)		P1 value	P2 value	P3 value
			%		%		%			
CRD-EPI	<90 mL/min/1.73 m^2^	8	23.53	12	25.53	-		>0.05		
	>130 mL/min/1.73 m^2^	1	2.94	5	10.64	-		>0.05		
MDRD	<90 mL/min/1.73 m^2^	12	35.29	18	38.30	-		>0.05		
	>130 mL/min/1.73 m^2^	1	2.94	4	8.51	-		>0.05		
CGF1	<90 mL/min	1	2.94	4	8.51	7	14.9	>0.05	>0.05	>0.05
	>130 mL/min	8	23.53	14	29.79	10	27.3	>0.05	>0.05	>0.05
CGF2	<90 mL/min	3	8.82	1	2.13	2	4.3	>0.05	>0.05	>0.05
	>130 mL/min	6	17.65	17	36.17	24	51.6	>0.05	<0.001	<0.05

P1 – difference in indicators between Groups 1 and 2; P2 – difference in indicators between Group 1 and Group 2 adjusted; P3 – difference in indicators between Group 2 and Group 2 adjusted

## Discussion

Assessing the body weight of an amputee has been a long-standing problem in medicine. It is difficult to weigh an amputee, and doctors often rely on the body weight before surgery, by subtracting the mass of the lost body part while using the previously proposed methods [14] which do not allow for an accurate assessment of the required value, since the patient may have a part of the limb that does not fit into the developed assessment. In addition, this approach does not provide a real assessment of the body weight, which may change over time. There is also the problem of assessing an excess body weight and obesity in a patient after the amputation [25]. Some authors recommend considering other techniques than BMI when assessing the metabolic status and the body composition of the patients after amputation, such as caliperometry, bioimpedancemetry, bone densitometry, and dual-energy X-ray absorptiometry [25,26]. In our opinion, although these methods are useful, they mostly require the use of additional resources and are therefore unlikely to become widely used. Perhaps, in this matter, research into individual 3D modeling and the capabilities of artificial intelligence for a more accurate assessment of the percentage of the lost body weight, with a focus on the adjusted BMI indicator, will be useful.

Creatinine is a product of muscle breakdown [27]; therefore, the level of this indicator in the blood can be falsely reduced in patients after a limb amputation, and the creatinine clearance can correspondingly increase [2,15,23,24]. Creatinine levels were significantly lower in the military personnel who underwent traumatic limb amputation than in the general population, as determined in a study conducted in the USA [23]. Such patients, as a rule, receive antibacterial drugs and/or opioids, and an inaccurate assessment of the kidney function based on the creatinine clearance can lead to incorrect medicine dose calculations in amputees [13,28].

Recently, the possibility of optimizing the kidney function assessment in amputees has been discussed in the literature [2,13,15,23,24,28,29]. For this purpose, researchers considered the need to introduce correction coefficients into GFR calculation formulas for this contingent as well as the choice of the optimal GFR calculation formula. Alternative biomarkers for a more accurate assessment of the renal clearance in amputees, particularly cystatin C, have also been studied [23,29].

The possibility of considering the lost body weight of amputees in the formulas for calculating GFR was studied by Im et al. [23] in a retrospective study involving 255 military personnel with traumatic amputations whose muscle mass loss was calculated according to a methodology developed by Osterkamp [14]. The authors studied the levels of serum creatinine and GFR calculated according to MDRD and CKD-EPI in groups of amputees as well as in a group of patients from the American *National Health and Nutrition Examination Survey* (NHANES) III cohort, selected by the sex, age, and race. The average serum creatinine level in the group of amputees was significantly lower than that in the population group (0.83±0.137 mg/dL vs. 1.14±0.127 mg/dL, p<0.0001). The percentage of the body weight lost correlated with the creatinine level and GFR calculated according to both formulas mentioned above [23].

Recently, certain limitations of the GFR calculation using MDRD and CKD-EPI in some categories of patients have been described in the literature. Thus, Pereverzeva et al. [30] recommend using the traditional methods – the Roberg-Tareev test or CGF – for the calculation of GFR in the course of the examination of young patients without a kidney pathology with stage 1/2 hypertension. This recommendation is related to the fact that, according to the data provided by these authors, new formulas for calculating GFR (MDRD and CKD-EPI) underestimate the results of determining the indicator in this contingent by 10.0–16.0% [30].

Similar data were obtained by Pertseva and Rokutova [31], who proved that the most informative methods for calculating GFR in young patients are the Roberg-Tareev test or CGF without correction for BSA. The MDRD and CKD-EPI formulas offer low informativeness in this population [31].

The plasma concentration of cystatin C, which is considered a new biomarker of glomerular filtration [15,29,32], seems to be completely dependent on GFR (freely filtered in the glomeruli, completely reabsorbed and catabolized in the proximal tubules, and tubular secretion is absent). It is also believed that the production of cystatin C does not depend on the presence of the age, sex, muscle mass, or the degree of hydration of the body. However, recent studies have shown that the concentration of cystatin C in plasma is still related to the subject’s age, BMI, sex, smoking status, and a high level of C-reactive protein and inflammation regardless of the renal function [33–36]. Recently, ALkhader et al. [36] found a strong positive correlation between cystatin C and the levels of such inflammatory factors as interleukin-6 (0.986, p=0.0001) and tumor necrosis factor-alpha (0.788, p=0.0001). Later, an immunoregulatory effect of cystatin C in macrophages was demonstrated [37].

Aakjær et al. [2] researched the effect of amputation due to nontraumatic causes on an estimated GFR based on creatinine and cystatin C in 38 patients (mean age 75 years) in a cohort study. After the amputation, GFR increased by 8.5 mL/min/1.73 m^2^ when calculating the indicator based on creatinine, by 6.1 mL/min/1.73 m^2^ when calculating based on cystatin C, and by 7.4 mL/min/1.73 m^2^ when calculating according to both indicators (in all cases, p<0.01). According to the data of these authors, a significant difference in the values of GFR when calculating based on creatinine, cystatin C, and both markers had a significant impact on the choice of the doses of drugs received by patients. Differences in doses occurred in 10.8–37.8% of patients, depending on the choice of the GFR calculation method (the largest difference was determined when calculating according to cystatin C), which created problems when choosing the doses of prescribed drugs that are excreted by the kidneys. In this study, such remedies included morphine and analgesics, an overdose of which could contribute to an increase in the number of undesirable effects, and, in the case of morphine, respiratory depression. The authors confirmed that the ‘gold standard’ and the most accurate methods for determining GFR are those that use exogenous markers (inulin and iohexol) [2].

In a secondary review of data from a retrospective cohort study by Iversen et al. [15], different approaches for determining GFR by using formulas based on creatinine, cystatin C, beta-trace protein, and beta-2-microglobulin were evaluated in patients who underwent extensive amputation. Twenty-nine patients (median age 75 years, 69% men) were included in the analysis. A significant decrease in the serum creatinine concentration (by 0.09 mg/dL, p=0.004) and an increase in GFR (by 6.1–6.3 mL/min, p=0.006) were detected in patients who underwent amputation. The authors concluded that creatinine-based estimates were more reliable than values based on cystatin C estimates and questioned the feasibility of using beta-trace protein and beta-2-microglobulin to estimate GFR in amputees [15].

According to our data, the calculation of GFR based on CGF, which considers the lost part of the body, is more in line with the pathogenetic features of amputees, considering also their inflammatory state [38] and smoking, which is popular among combatants [39]. The final two options can influence the cystatin C levels. Persistence of the systemic inflammatory response syndrome in patients after a severe combat injury can lead to an early cardiovascular pathology manifestation [39]. In the course of conducting the PATRIOT project, we found that combatants, after a combat injury, including those with amputations, had signs of sustained inflammation even after 45 days after the trauma, with worse parameters being observed in amputees [38]. Finally, we were interested in the methods available at all military medical support levels, since cystatin C measurement may be unavailable/problematic even on the level of Military Medical Clinical Centers in Ukraine.

In our opinion, another question can be discussed and further investigated: How can we adjust the creatinine serum levels in amputees depending on the lost body part? In our study, we used, as other researchers had done before us, the adjusted body weight, but not creatinine levels. This question still needs careful investigation.

Using CGF with an adjusted body weight/BSA makes it possible to avoid the underestimation of indicators by the MDRD and CKD-EPI formulas in young people, which was also demonstrated in our study, as well as to identify patients with the hyperfiltration syndrome. It is generally accepted that renal hyperfiltration is a phenomenon in which GFR values are above the 95^th^ percentile in a healthy population with fluctuations within two standard deviations above the mean [21,40], but such a definition can be confusing for practical doctors, and therefore we used a more practical approach and defined renal hyperfiltration in the case of the GFR value measuring >130 mL/min/1.73 m^2^ [20]. In the literature, a GFR value above 130–135 mL/min/1.73 m^2^ is usually considered renal hyperfiltration [20,40,41].

Glomerular hyperfiltration, regardless of etiology, is considered an early stage of chronic renal failure, and it usually precedes the occurrence of microalbuminuria. The development of hyperfiltration is explained by intraglomerular hypertension (an increase in the level of hydrostatic pressure in the glomerulus) due to an imbalance in the tone of the afferent and efferent arteries – a decrease in the tone of the afferent artery with a relative predominance of the tone of the efferent artery. The role of intrarenal hemodynamic disorders in the development of hyperfiltration has been discussed in the literature since the end of the 20^th^ century, but, even today, this phenomenon has not been sufficiently explored, and the criteria for its diagnosis have not been developed yet.

Recently, it has been shown that glomerular hyperfiltration is independently associated with an increased cardiovascular risk in middle-aged healthy individuals. In the research conducted by Dupuis et al. [21], the authors found a cardiovascular risk profile to be comparable to that of patients with GFR between 45 and 60 mL/min/1.73 m^2^. Moreover, in the most recent publications, an association between renal hyperfiltration and non-cardiovascular smoking-related mortality has been found [42].

Hyperfiltration is best studied in patients with diabetes. These studies began in 1998, after Chaiken et al. [43] showed that hyperfiltration was present in 36% of patients with type 2 diabetes in whom the disease was diagnosed within a year and persisted for 10 years in 14–20% of the patients.

A retrospective study of 314 patients with a long course of type 1 or 2 diabetes was conducted by Moriconi et al. [44], in which glomerular filtration was determined by using dynamic scintigraphy with 99mTc-DTPA; not only hypofiltration but also hyperfiltration contributes to an increase in the combined endpoint (the risk of deterioration of the renal function and mortality from cardiovascular and renal causes) in patients with diabetes. There are assumptions about the role of ultrastructural (an increase in the kidney size and the filtration surface area), vascular (imbalance of vasoactive humoral factors that control the tone of pre- and postglomerular arterioles), and tubular (violation of interaction between glomeruli and tubules, increased reabsorption of sodium and glucose in the proximal tubules as a cause of a decrease in the resistance of afferent arteries) factors in the pathogenesis of hyperfiltration in patients with diabetes [45].

Anyway, Ould Setti et al. [46] showed that the association between renal hyperfiltration and mortality was not mediated by diabetes mellitus. Based on their findings, the authors declared that clinicians should consider screening, follow-up and managing renal hyperfiltration as a mortality-associated condition regardless of the presence of diabetes [46].

Currently, the data that have been accumulated are still insufficient regarding the development of generally accepted recommendations for the correction of the dose of drugs excreted by the kidneys in amputees. This is primarily due to the lack of studies of GFR in amputees based on the ‘gold standard’ – the determination of GFR by using exogenous markers. Currently, the recommendations of Elbarbry et al. [13] are based on the analysis of data from the literature and the results of the authors’ own research:

- In cases of prescribing drugs which do not require dosing depending on the body weight and the kidney function, or in cases with prescription of drugs based on the body weight with a wide therapeutic index, the dose may be usual (or slightly reduced) [47];
- In cases of prescribing drugs with a narrow therapeutic index, or those with an unfavorable toxicity profile, there is a need to consider the GFR calculation with correction for the lost body weight [13].

The therapeutic index is the standard pharmacological designation of the dose range at which the medicine remains both effective and safe without unacceptable adverse events. A narrow therapeutic index means a small range of effective doses without adverse toxic effects [48]. Several medicines with a narrow therapeutic index can be prescribed to the injured, including amputees [13], and thus there is a need to control more carefully their GRF so that to prescribe a safe dose of the medicine.

However, the study of therapeutic support specifics for patients with a combat limb trauma involving amputation requires the planning of a series of well-designed studies that can provide answers to the relevant questions which arise in the real-world clinical practice.

The present study had certain limitations. We included only men in the study, which was due to the extremely rare cases of limb amputation due to a combat trauma in women. Thus, this limits the conclusions of the study to the male population. In addition, the study included patients who underwent amputation of the upper or lower limbs at different levels, and patients who underwent the amputation of more than one limb. Such a design has both advantages (assessments under real-world conditions) and limitations (the degree of severity of metabolic disorders and the loss of the body weight may differ significantly). We did not evaluate GFR by using ‘gold standard’ methods with exogenous markers, as the goal of this study was to develop approaches for practicing clinicians based on the methods available in the medical facilities of the Ministry of Defense of Ukraine at most levels of medical care. We also did not analyze 24-hour proteinuria/albuminuria data, including albumin/creatinine or protein/creatinine ratio as it was not the routine test for the protocol of management of the patient with combat-isolated injury to limbs. Internal pathology (including kidney disease) was an exclusion criterion for the patient selection; no protein was detected in a single portion of urine in the examined patients.

## Conclusions

Thus, our analysis has revealed several features that must be considered in the management of combat limb injury patients, including amputees:

- The need to calculate the adjusted body weight of amputees considering the lost body part for an adequate assessment of their metabolic status as well as the need to develop new techniques for more precise individual assessment of the metabolic status and the body composition of the amputee;
- The expediency of using CGF for the calculation of GFR in the amputee, considering the loss of the body weight with/without a correction for BSA for proper medicine dose adjustment;
- If only creatinine-based techniques are available for GRF assessment in the amputee, it may be necessary to develop principles for correcting the creatinine level for the lost part of the limb to the ‘ideal’ level in order to be able to compare the value with that of patients without amputation for the correct determination of renal dysfunction according to the currently approved classification;
- A careful follow-up and further examination are necessary for the injured individuals in whom renal hyperfiltration is suspected when calculating GFR based on CGF with/without a correction for BSA, as this condition can be a predictor of future metabolic disorders.
